# Supplementary figures and images for: Changes in mobility patterns during the COVID-19 pandemic in Zambia: Implications for the effectiveness of NPIs in Sub-Saharan Africa
Source: PLOS Glob Public Health. 2023 Oct 31;3(10):e0000892. doi: 10.1371/journal.pgph.0000892 (PMC10617722; doi:10.1371/journal.pgph.0000892)

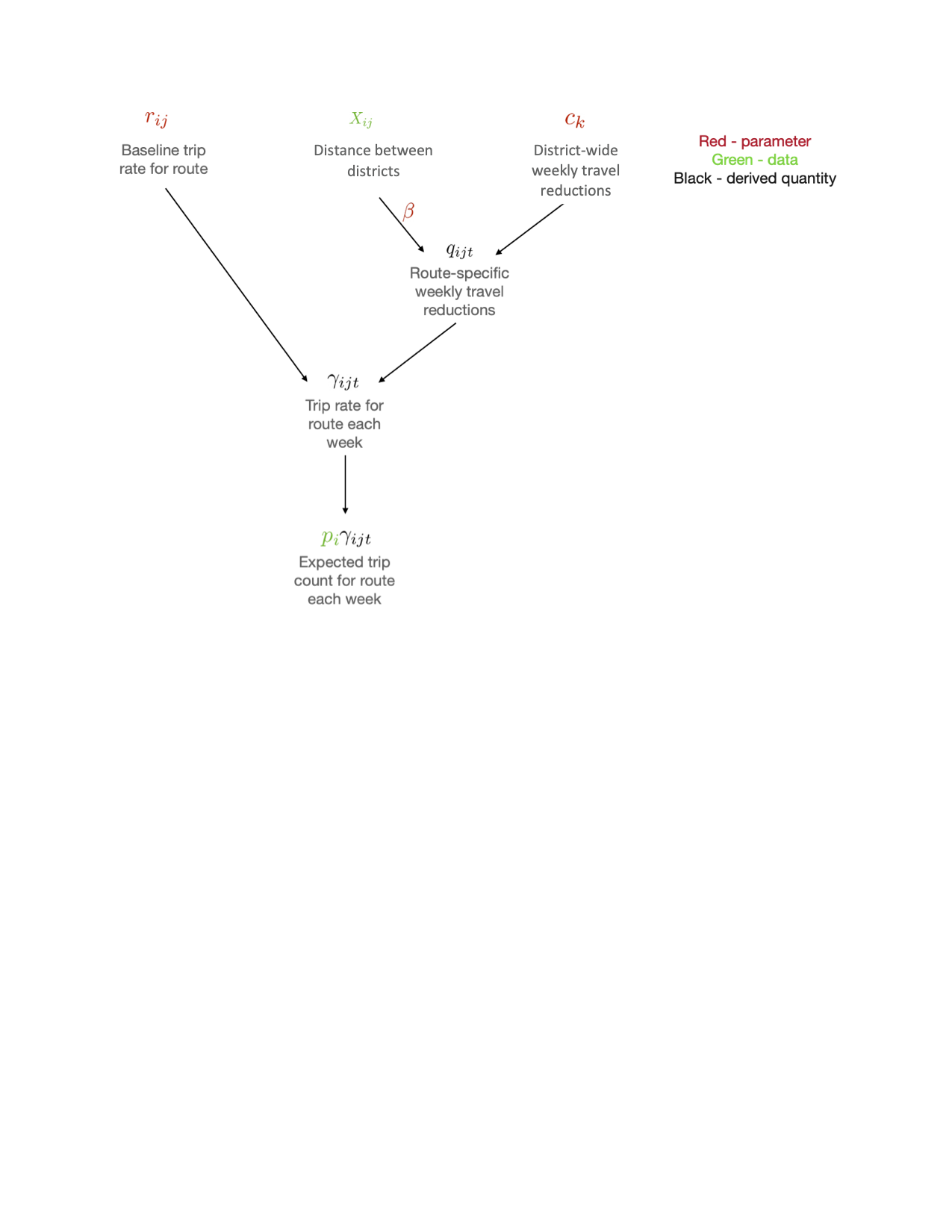

Supplement: S1 Fig — Visualization of the mobility model used in this analysis. (TIFF) [file pgph.0000892.s001.tiff]

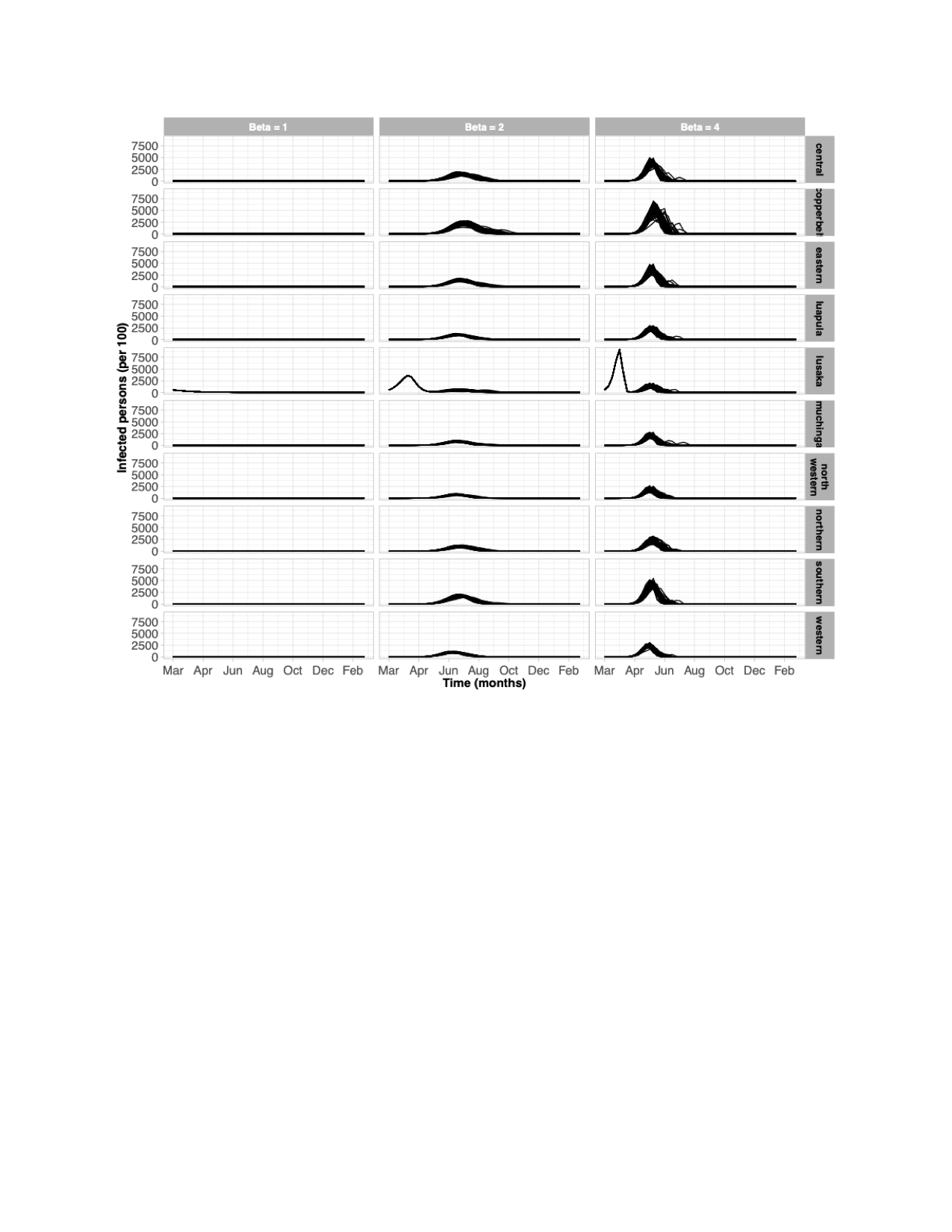

Supplement: S2 Fig — We varied the transmission coefficient (β0) to determine sensitivity of case distribution. We ran the model with β0 = 1,2, and 4. The transmission coefficient should be approximated by R and β0 = 1 and β0 = 4 results in lower and higher transmission and case incidence then what has been shown, respectively. (TIFF) [file pgph.0000892.s002.tiff]

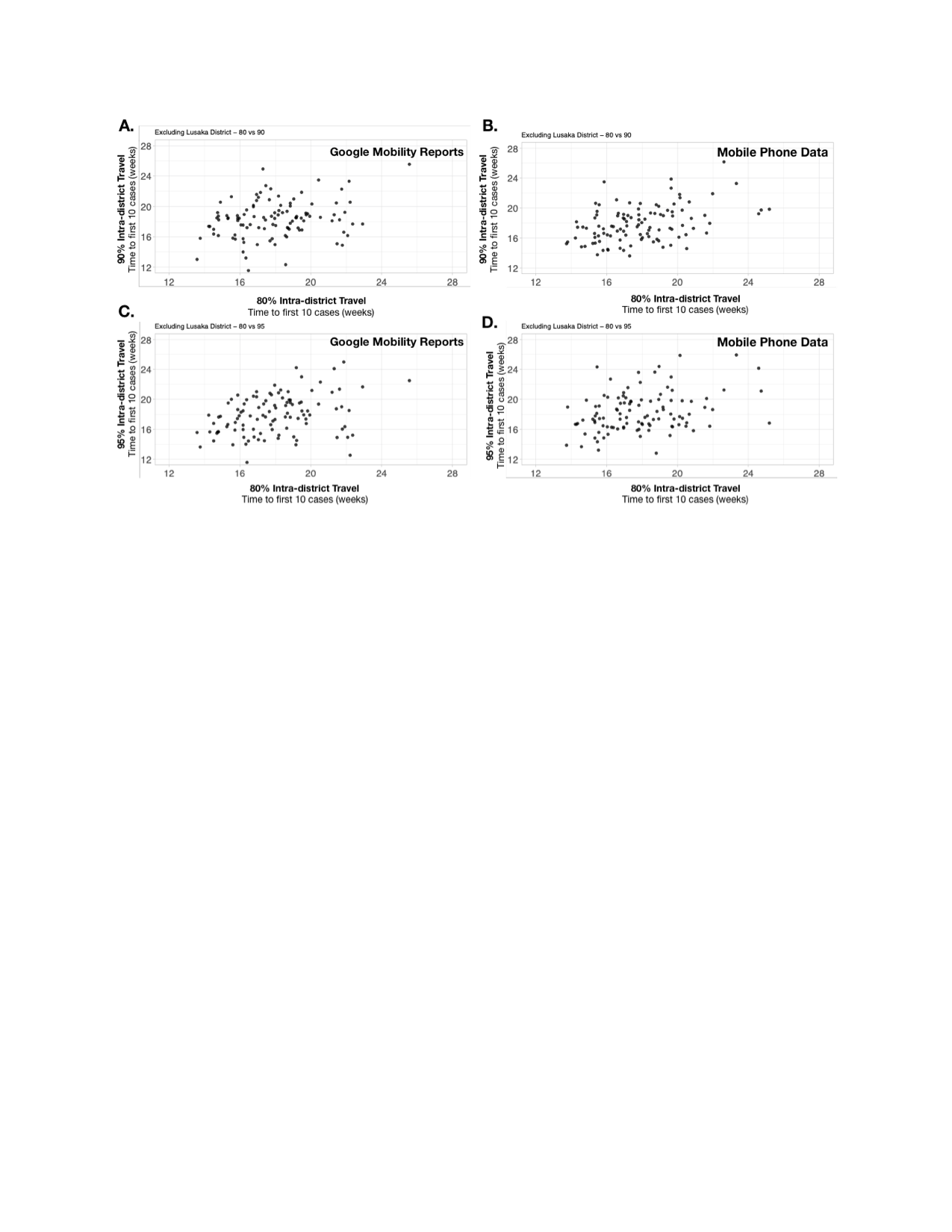

Supplement: S3 Fig — Time to first 10 cases comparing 80% versus 90% intra-district travel for (A) Google Mobility Reports and (B) mobile phone data. Time to first 10 cases comparing 80% versus 95% intra-district travel for (C) Google Mobility Reports and (D) mobile phone data. Since cases were introduced in Lusaka District in the transmission model, all outputs exclude Lusaka District. (TIFF) [file pgph.0000892.s003.tiff]

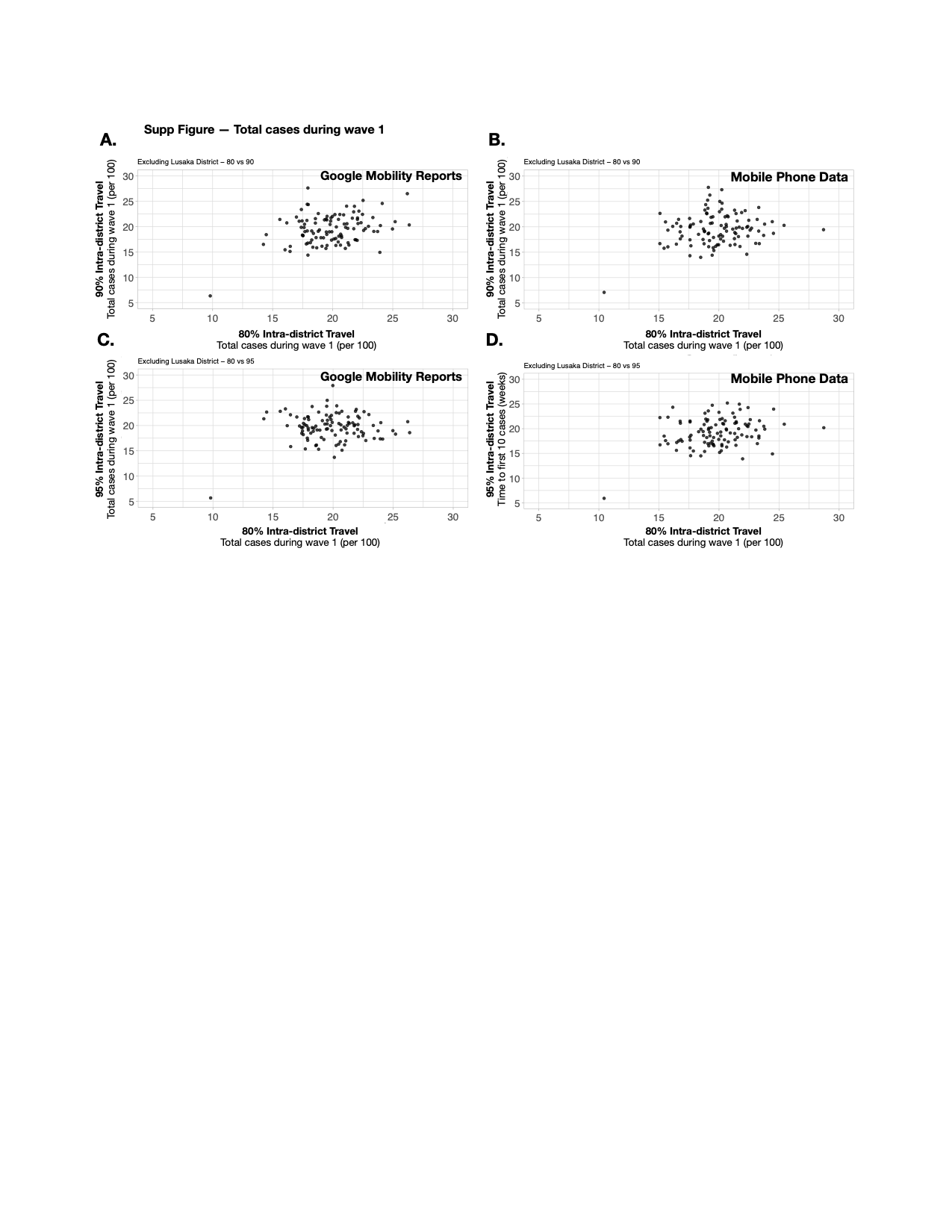

Supplement: S4 Fig — Total cases during wave 1 comparing 80% versus 90% intra-district travel for (A) Google Mobility Reports and (B) mobile phone data. Total cases during wave 1 comparing 80% versus 95% intra-district travel for (C) Google Mobility Reports and (D) mobile phone data. Since cases were introduced in Lusaka District in the transmission model, all outputs exclude Lusaka District. (TIFF) [file pgph.0000892.s004.tiff]

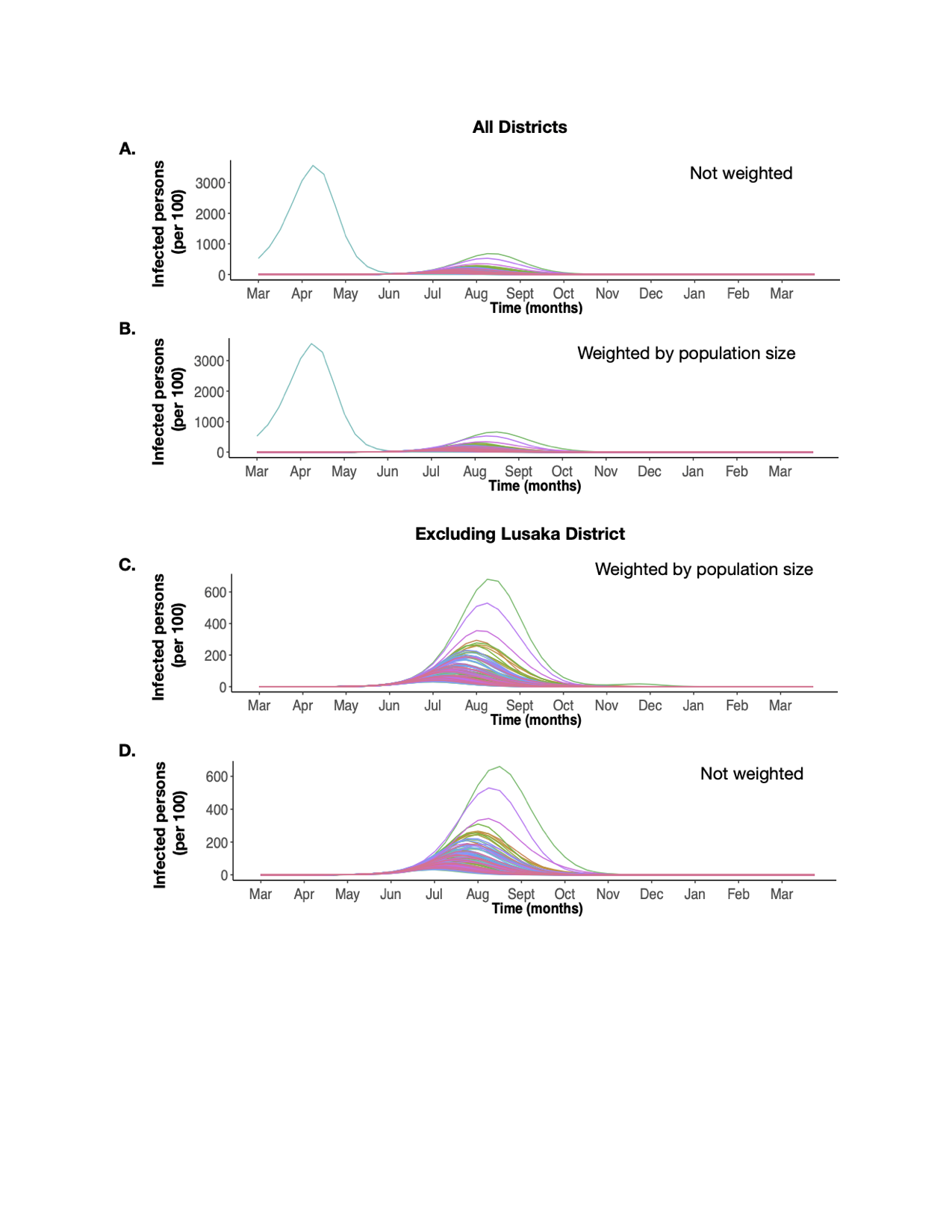

Supplement: S5 Fig — (A). Time series of infected persons without using any weights and only using aggregated trip averages with Lusaka District included. (B) Time series of infected persons with district population size as weights for trips with Lusaka District included. (C) Time series of infected persons without using any weights and only using aggregated trip averages with Lusaka District excluded. (D) Time series of infected persons with district population size as weights for trips with Lusaka District excluded. (TIFF) [file pgph.0000892.s005.tiff]

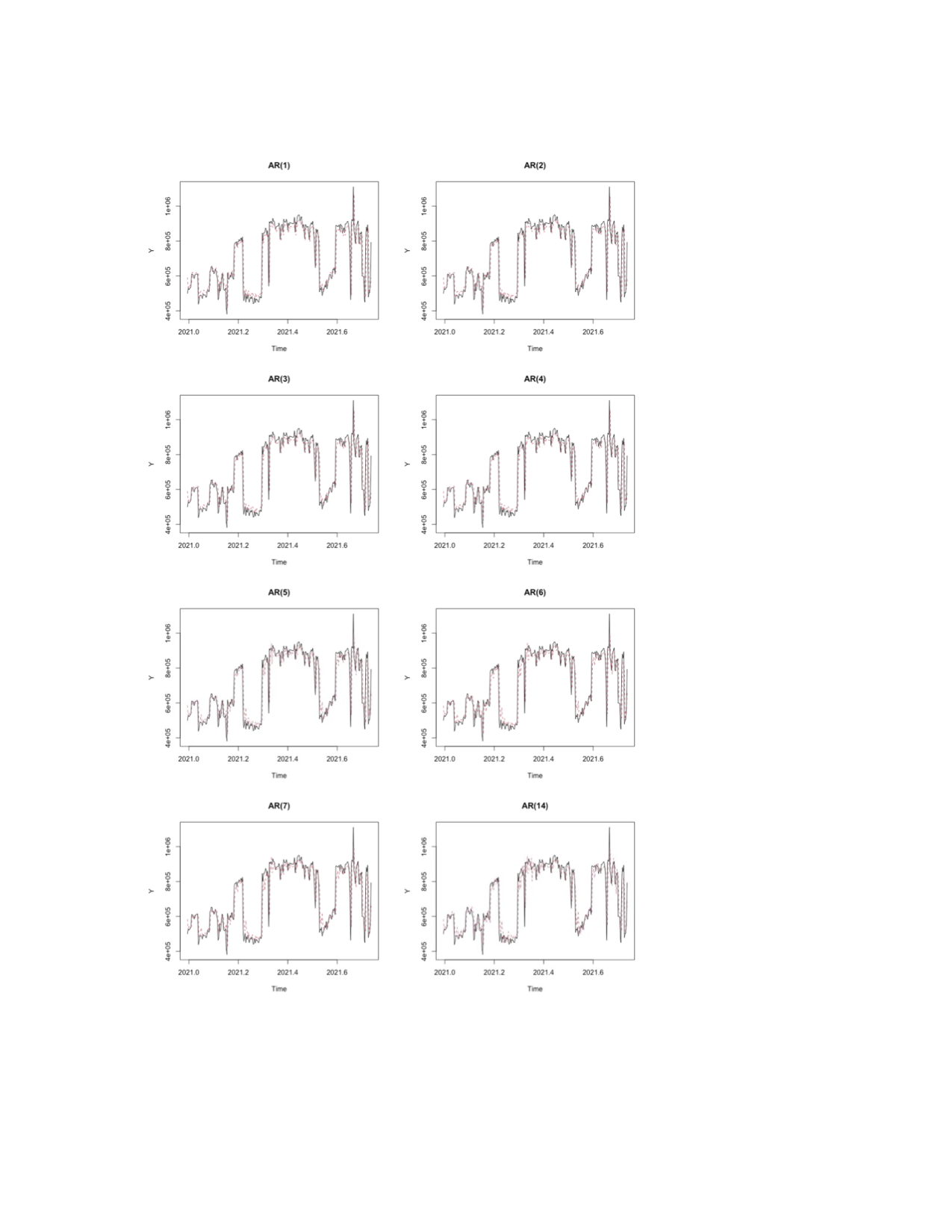

Supplement: S6 Fig — Lags of 1–7 and 14 were explored to assess autocorrelation. The Y-axis are the total number of national trips per day and the x-axis is time in days. (TIFF) [file pgph.0000892.s006.tiff]

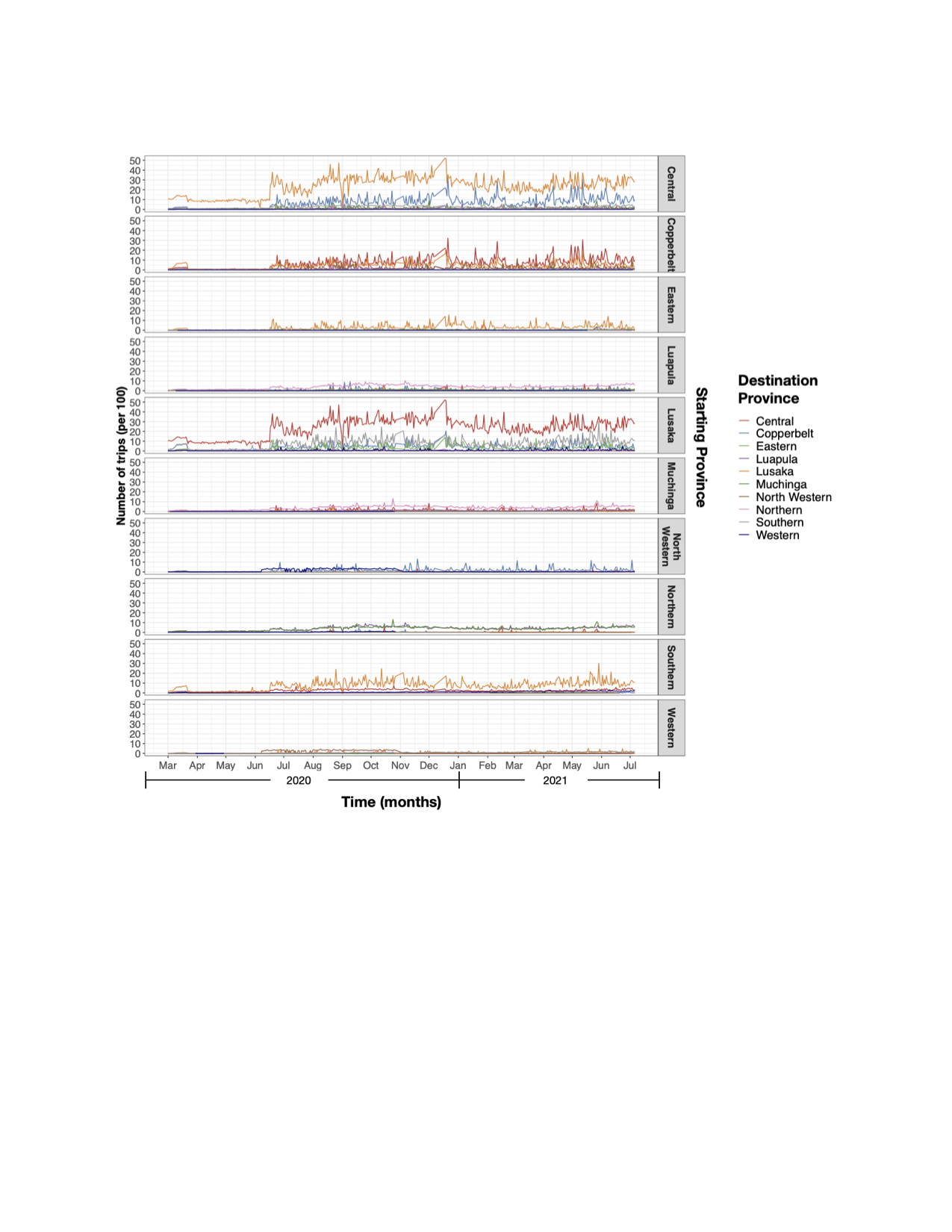

Supplement: S7 Fig — Each row represents the starting province and each line represents the total number of daily trips to a destination province. Intra-province trips (trips occurring within the same province) are excluded from this figure. (TIFF) [file pgph.0000892.s007.tiff]

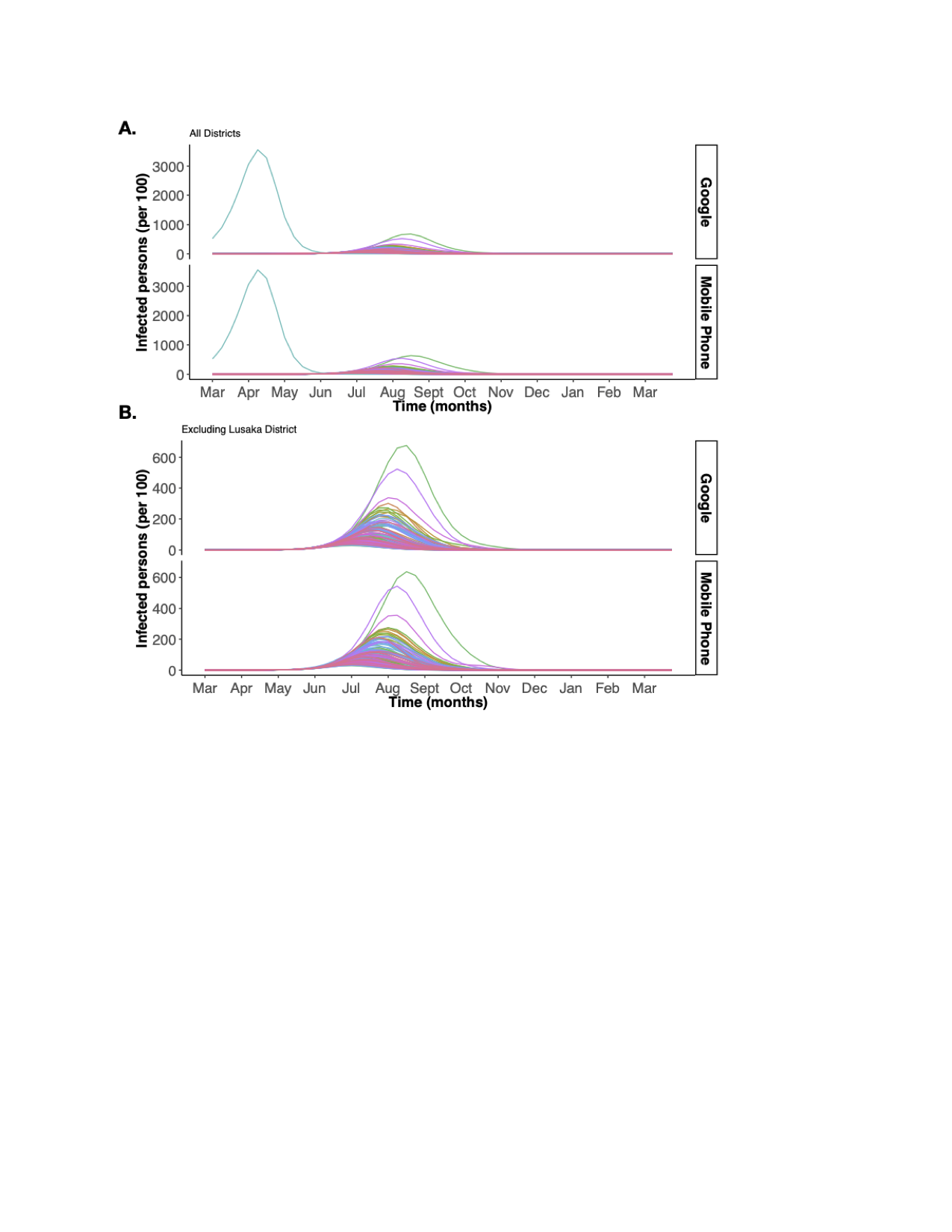

Supplement: S8 Fig — (A) Time series of infected persons using Google versus Mobile Phone data with Lusaka District (blue-green line) included. (B) Time series of infected persons using Google versus Mobile Phone data excluding Lusaka District. (TIFF) [file pgph.0000892.s008.tiff]

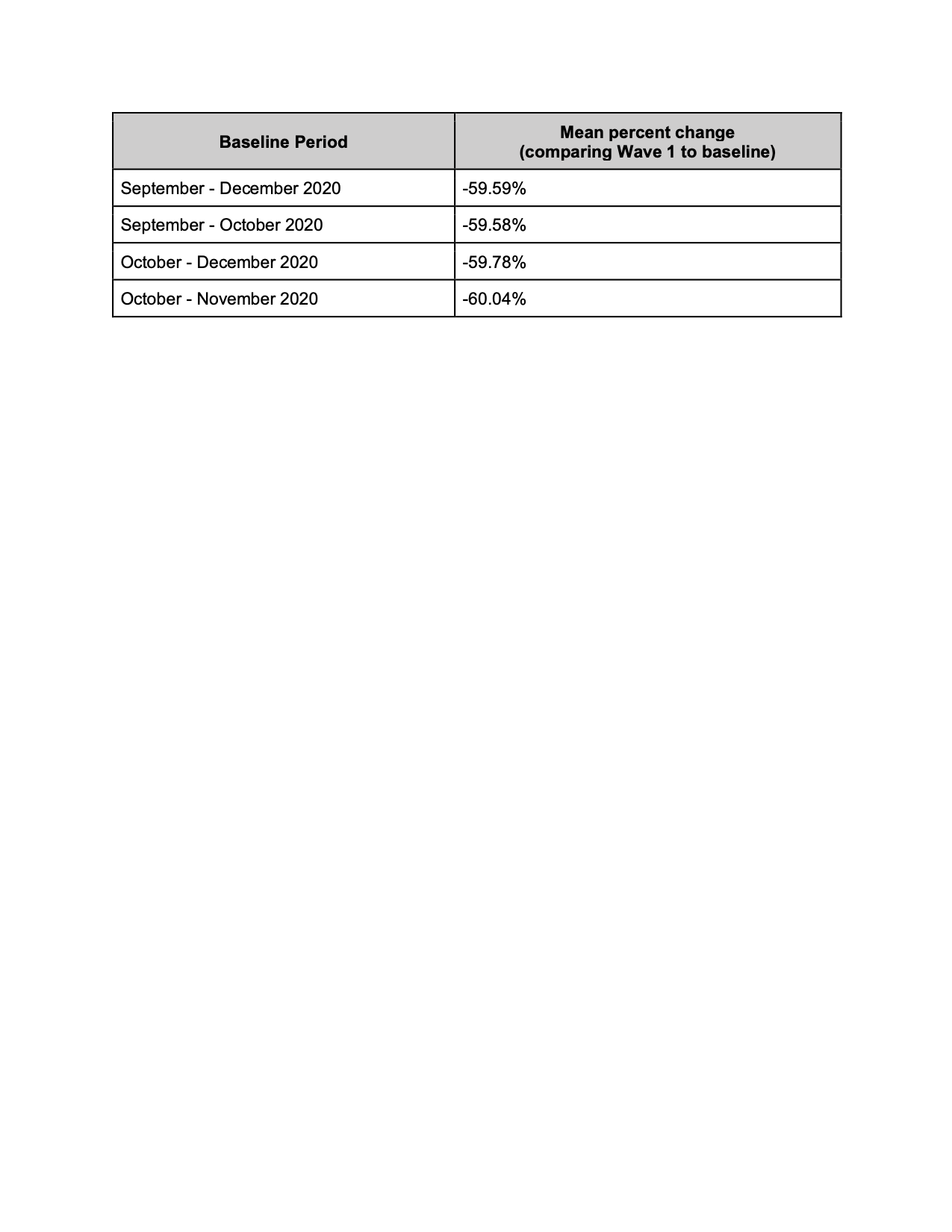

Supplement: S1 Table — Different variations of baseline were explored to see if there were any notable quantitative differences between variations. The percent changes reflect the percent change on overall mobility comparing Wave 1 to baseline. (TIFF) [file pgph.0000892.s009.tiff]

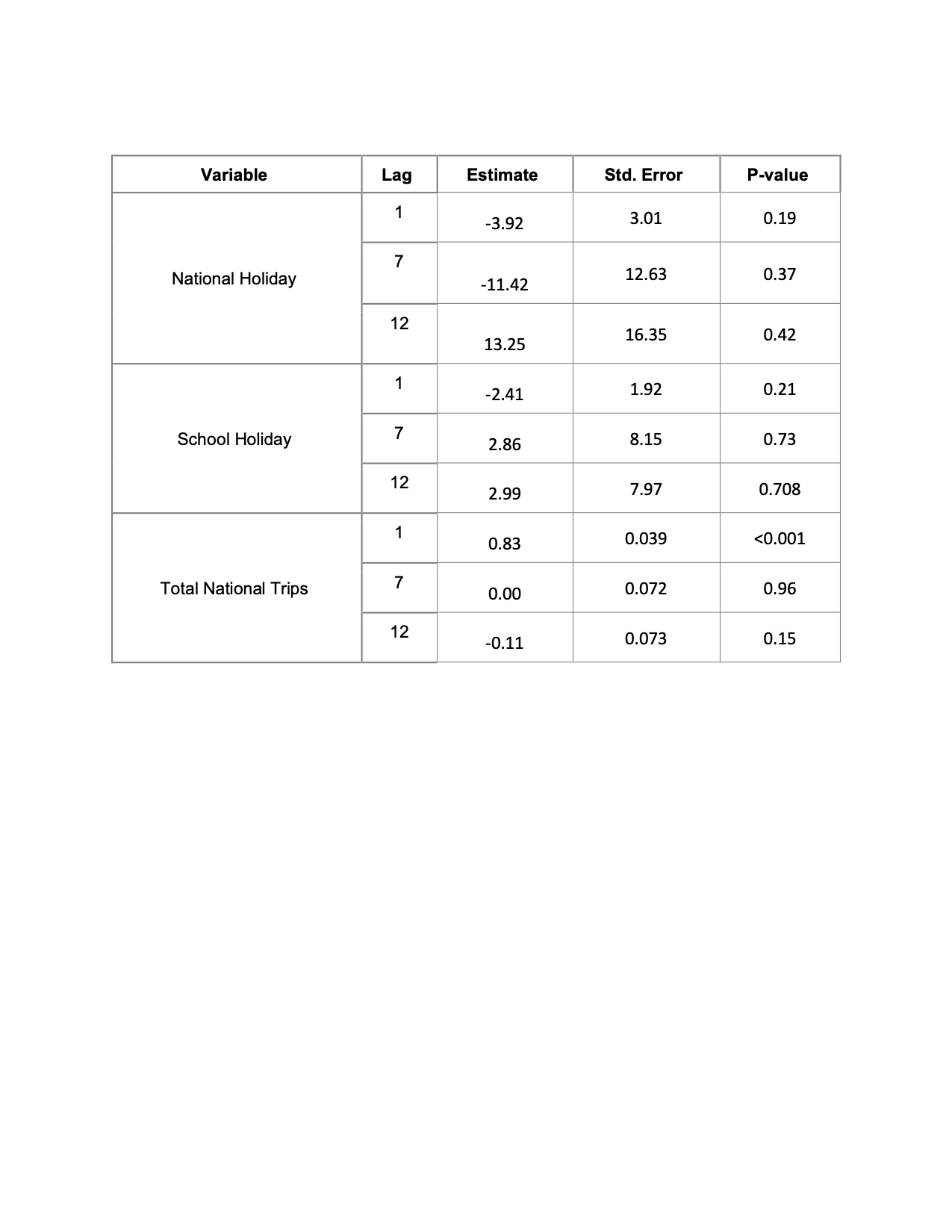

Supplement: S2 Table — To account for temporal autocorrelation, three different lags representing days: 1, 7, and 12 were considered in this analysis. National or school holidays consistently were not significantly associated with changes in travel. (TIFF) [file pgph.0000892.s010.tiff]
